# Exploring Matrix Stiffness‐Related Gene in Periodontitis: A Comprehensive Multidataset Analysis

**DOI:** 10.1155/mi/2009746

**Published:** 2026-08-26

**Authors:** Yanhui Peng, Xiaoqin Yang, Wei Fang, Lifang Zhang, Ting Guo, Hongyu Zheng

**Affiliations:** ^1^ Stomatological Hospital, Southern Medical University (Guangdong Provincial Stomatology Hospital), No. 366 South Jiangnan Avenue, Haizhu District, Guangzhou 510280, China; ^2^ The First Affiliated Hospital of Zhengzhou University, No. 1 Jianshe East Road, Erqi District, Zhengzhou 450052, Henan, China, zzu.edu.cn

**Keywords:** biomarkers, machine learning, matrix stiffness, periodontitis

## Abstract

**Background:**

Characterized by the progressive deterioration of periodontal supporting tissues, periodontitis (PD) is a prevalent oral disorder primarily instigated by dental plaque biofilms. Matrix stiffness (MS), a key extracellular matrix (ECM) property, regulates inflammatory cell function and tissue repair balance. Herein, we sought to screen for potential biomarkers linking MS to PD pathogenesis and characterize the molecular mechanisms governing their regulation.

**Methods:**

To detect robust biomarkers, we utilized differential expression analysis and machine learning approaches (LASSO, random forest, and Boruta), followed by rigorous validation. Beyond biomarker identification, the study involved functional enrichment, immune infiltration estimation, drug screening, and regulatory network mapping, all integrated with single‐cell data.

**Results:**

Four upregulated MS‐related biomarkers (COL15A1, COL4A1, CXCR4, and MMP7) were identified, enriched in chemokine signaling and natural killer (NK) cell cytotoxicity pathways. Immune infiltration revealed 15 differentially abundant cell types (e.g., neutrophils and resting NK cells). Regulatory networks (e.g., MALAT1‐hsa‐miR‐124‐3 p‐COL4A1) and potential drugs (e.g., dipyridamole) were predicted. Single‐cell analysis identified 13 cell types, with endothelial cells showing high activity in fatty acid metabolism pathways.

**Conclusion:**

COL15A1, COL4A1, CXCR4, and MMP7 were identified as biomarkers associated with MS. Bioinformatics analysis suggested that they might be involved in the progression of PD through pathways such as immune infiltration, metabolism, and inflammatory chemotaxis, providing hypothetical clues regarding the link between MS and the pathogenesis of PD; however, their specific mechanisms of action still required experimental validation.

## 1. Introduction

Widely recognized as a chronic inflammatory condition driven by dental plaque biofilms, periodontitis (PD) is fundamentally characterized by a dysregulated host immune response that precipitates the progressive loss of periodontal connective tissue attachment and alveolar bone resorption [1, 2]. The occurrence and progression of this disease are closely associated with the complex interactions among bacterial infection, host immune response, and environmental factors [3]. Currently, clinical management primarily relies on mechanical therapeutic approaches such as supragingival scaling and subgingival scaling and root planning (SRP) to control the condition; however, the disease recurrence rate remains persistently high [4]. This is because the functions of particular genes, cellular subsets, and cellular regulatory mechanisms associated with its pathogenic processes remain poorly defined, and there are as yet no valid preclinical diagnostic biomarkers or therapeutic molecular targets for these inflammatory pathologies [5]. In this context, beyond traditional microbiological factors, emerging evidence highlights the critical role of the local physical microenvironment in disease pathogenesis. Specifically, matrix stiffness (MS)—a key biomechanical property of the periodontal extracellular matrix (ECM)—has been identified as a potent mechanical cue. Pathological alterations in MS can directly regulate cellular behaviors and immune cell activation, thereby exacerbating the inflammatory cascade.

The ECM acts as the principal extracellular constituent of bodily tissues and organs, conferring a structural scaffold and mechanical support to cellular populations. MS, a critical physical property of the ECM, is defined as the mechanical capacity of the matrix to resist external force‐induced deformation. It can regulate core biological behaviors of cells (including cell spreading, proliferation, and differentiation) through mechanotransduction pathways [6, 7]. In recent years, escalating interest has been centered on the functions and roles of MS in various diseases [8–12]. Watcharaphol et al. [13] demonstrated that decreased MS can bidirectionally promote fibroblast activation and the release of inflammatory cytokines such as IL‐1β and PGE_2_ by activating the NF‐κB and YAP signaling pathways, and these factors are precisely the key mediators that exacerbate periodontal tissue destruction.

Accumulating evidence has verified that MS is capable of modulating the osteogenic differentiation of human periodontal ligament stem cells (hPDLSCs) through the DNA methylation machinery [14]; concurrently, within a three‐dimensional hydrogel culture system, MS also serves as a pivotal biophysical regulatory factor that governs the osteogenic differentiation potential of hPDLSCs, with this regulatory effect being mediated by the YAP signaling cascade. Contrary to traditional perceptions, a moderately low‐stiffness microenvironment is more conducive to the osteogenic differentiation potential of hPDLSCs and bone tissue regeneration, which provides a novel research perspective and therapeutic strategy for the fields of periodontal tissue engineering and bone regenerative medicine [15].

Additionally, other studies have indicated that MS, as a key mechanotransduction factor, can influence the osteogenic differentiation potential of hPDLSCs by regulating the morphological characteristics, distribution, and functional homeostasis of mitochondria, thereby playing a crucial role in the clinical practices of oral implantation and orthodontics [16]. Drawing on the robust evidence outlined in the foregoing studies concerning the interplay among MS, PD, and the differentiation of periodontal ligament stem cells, we reasonably hypothesize that there is an inherent link between MS and the pathogenesis of PD.

Although previous studies have separately investigated the molecular characteristics of MS and PD, no integrated analysis has yet been reported that combines MS‐related gene sets with PD multiomics data—including bulk and single‐cell transcriptomics—and employs multiple machine learning algorithms to cross‐screen candidate genes. Therefore, this study aims to preliminarily identify MS‐related biomarkers through bioinformatic analysis of integrated multiomics datasets and to explore their potential expression profiles and immune associations in PD, thereby providing a hypothetical basis and preliminary clues for subsequent mechanistic studies and personalized therapeutic strategies.

## 2. Materials and Methods

### 2.1. Data Acquisition

PD‐related transcriptomic datasets were obtained from the GEO database (https://www.ncbi.nlm.nih.gov/geo/), which is maintained by the NCBI. GSE16134 (GPL570) was the training set, comprising 241 PD gingival tissues and 69 healthy age‐matched controls. For external validation, 10 PD gingival tissue samples were retained from GSE223924 (platform: GPL24676), whereas other samples were excluded. GSE171213 (platform: GPL24676) was used for single‐cell analysis after the exclusion of low‐quality specimens with incomplete clinical annotations. The dataset comprised periodontal samples from five PD patients and four controls. A total of 1417 MS‐RGs were sourced from the literature [17]. (Table S1).

### 2.2. Acquisition of Candidate Genes

Bulk DEGs in GSE16134 between PD and control gingival tissues were identified using limma (v3.56.2) [18]. The thresholds were |log_2_FC| > 1 and corr. *p*  < 0.05. The global DEG distribution was displayed with ggplot2 (Version 3.5.1) [19]. The top 10 upregulated and downregulated genes were annotated by the log_2_FC. Expression profiles of the top 10 genes in each direction were visualized using ComplexHeatmap (Version 2.1.6.0) [20].

Candidate genes were derived from bulk‐DEG ∩ MS‐RG intersections using VennDiagram v 1.7.3 [21], followed by GO/KEGG enrichment (clusterProfiler v4.15.0.3 [22], *p*  < 0.05). For visualization, the five terms with the smallest *p* values in each GO/KEGG category were selected. Proteins encoded by candidate genes were submitted to the STRING platform for PPI network construction, retaining interactions with a confidence score > 0.4.

### 2.3. Biomarkers Acquisition

The bioinformatic analysis workflow in this study was adapted from the methodology described by Xu et al. [23]. Three machine learning approaches were applied to the candidate gene set in GSE16134. LASSO regression was first conducted with glmnet (Version 4.1.8) [24] after setting the random seed to 74, and genes retained at the optimal lambda were recorded as LASSO genes. RF analysis was then implemented with randomForest (Version 4.7) [25], with the random seed set to 1 and ntree set to 13. Tenfold cross‐validation was used to select the top 10 RF feature genes. Boruta analysis was performed with Boruta (Version 8.0.0) [26] under a random seed of 4, and genes marked as “confirmed” were considered Boruta genes. VennDiagram (Version 1.7.3) was used to intersect LASSO, RF, and Boruta outputs and identify candidate biomarkers. Expression differences between PD and controls in GSE16134 and GSE223924 were assessed by Wilcoxon rank‐sum (*p*  < 0.05); genes showing consistent direction across both sets were kept as final biomarkers. The diagnostic performance in GSE16134 was assessed with pROC (Version 1.18.5) [27] by generating ROC curves and calculating AUC values. Biomarkers with an AUC > 0.7 were considered to show acceptable predictive ability.

### 2.4. Nomogram Construction and Assessment

A biomarker‐based nomogram for PD prediction was built in GSE16134 using rms (Version 6.8.1) [28]. Calibration was evaluated with regplot (v1.1.0) [29] by drawing the calibration curve and applying the Hosmer–Lemeshow (HL) test. Calibration agreement was acceptable at *p*  > 0.05; clinical utility was evaluated via DCA using rmda v1.6 [30].

### 2.5. Investigation of Signaling Pathways Related to Biomarkers

GSEA used MSigDB c2.cp.kegg.v7.4.symbols.gmt and clusterProfiler v4.15.0.3. Correlation coefficients between each biomarker and all GSE16134 genes were computed with psych v2.4.6.26 [31]. Genes were ordered from the highest to the lowest correlation coefficient to generate biomarker‐specific ranked lists. Pathways with |NES| > 1 and adj. *p*  < 0.05 were retained; the 10 lowest‐adj. *p* pathways per biomarker were displayed.

### 2.6. Immune Microenvironment Analysis

Relative abundances of 22 immune cell subsets in GSE16134 were estimated using the CIBERSORT algorithm [32]. Samples with CIBERSORT *p*  > 0.05 were excluded. Wilcoxon rank‐sum (*p*  < 0.05) tested immune cell fraction differences, followed by Spearman correlations (psych v2.4.6.26) between biomarkers and differential immune subsets. Associations meeting |cor| > 0.30 and *p*  < 0.05 were retained, and the five strongest correlations ranked by |cor| were visualized.

### 2.7. Exploring Biomarker Functions From Multiple Perspectives

The genomic positions of the biomarkers were visualized with RCircos (Version 1.2.2) [33]. Subcellular localization data for each biomarker were obtained from the compartments Database.

DIANA‐microT and miRDB predicted biomarker‐targeting miRNAs; overlapping miRNAs from both databases were considered key regulatory miRNAs. Upstream lncRNAs interacting with miRNAs were searched in starBase (human, CLIP‐Data ≥ 4); TFs were sourced from KnockTF. lncRNA–miRNA–mRNA and TF‐mRNA networks were then built and visualized to summarize the predicted regulation.

### 2.8. Drug/Compound Prediction

DGIdb was searched to identify drugs or compounds with predicted interactions with the selected biomarkers. A drug/compoundmRNA network was then generated to depict these predicted relationships.

### 2.9. Single‐Cell Analysis

Single‐cell RNA‐seq data from GSE171213 were analyzed with Seurat (Version 5.0.1) [34] under stringent QC settings. Genes present in at least five cells were kept, along with cells having >200 genes. Further filtering: nFeature RNA was restricted to the range of 200–6000, nCount_RNA < 20,000, and percent.mt < 40%.

After QC, the expression matrices were normalized using LogNormalize. The FindVariableFeatures function was applied to select the top 2000 HVGs, and LabelPoints was used to annotate the top 10 HVGs in the plot. PCA was performed on HVGs with RunPCA. Informative PCs were selected according to the inflection point in the ElbowPlot to preserve major biological variation while limiting noise.

Batch effects were adjusted with RunHarmony from the Harmony package (Version 1.2.3) using PCA‐derived embeddings. Based on Harmony‐corrected reductions, FindNeighbors and FindClusters were used for clustering. UMAP was then performed with the same reduction at a resolution of 0.1. Cell identities were assigned according to established marker genes reported previously [35], and marker expression across annotated cell types was presented in a bubble plot.

Pathway enrichment per annotated cell type in GSE171213 was performed with ReactomeGSA v1.16.1 [36]. The minimum enrichment score across all pathways was treated as the reference baseline. For each pathway, the enrichment score and its deviation from this baseline were calculated. Pathways were ranked by deviation, and the 10 pathways with the largest deviations were displayed.

### 2.10. Identification of Key Cells and Pseudotime Analysis

Histogram plots compared cell‐type composition between PD and controls; Wilcoxon rank‐sum tests (*p*  < 0.05) identified significantly different proportions. Bubble plots showed biomarker expression across cell types; cells with both significant proportion changes and multibiomarker differential expression were chosen as key cells.

For the key cell population, dimensionality reduction and clustering followed the workflow described in Section 2.9, with the resolution fixed at 0.1. Key cells were further divided into subclusters using validated marker genes from prior studies [35, 37]. Pseudotime trajectories were reconstructed with Monocle (Version 2.28.0) [38] to characterize the differentiation status and developmental progression. Changes in target biomarker expression along pseudotime were evaluated to identify potential regulatory stages during cell‐state transition.

### 2.11. Cell Communication, Cycle, and Metabolism Analysis

Intercellular communication among predefined key cell subsets in GSE171213 was inferred with CellChat (Version 1.6.1) [39]. Candidate ligand–receptor interactions between cell types were quantified and screened to characterize signaling crosstalk.

Cell‐cycle scores and phase assignments were calculated using CellCycleScoring. Stacked bar plots were used to show the distribution of cell‐cycle phases in PD and control groups. Wilcoxon rank‐sum tests compared cell‐phase proportions; *p*  < 0.05 was considered significant.

The scMetabolism package (Version 0.2.1) [40] was applied to calculate metabolic pathway activity scores within the key cell population. This analysis enabled the pathway‐level evaluation of metabolic alterations.

### 2.12. Immunohistochemistry (IHC)

IHC staining was conducted on 10 PD tissues and 10 healthy control tissues collected in this study to verify COL4A1, CXCR4, and MMP7 protein levels. The study followed the Declaration of Helsinki and received approval from the Ethics Committee for Scientific Research Projects, The First Affiliated Hospital of Zhengzhou University (Approval Protocol Code: 2023‐KY‐1160‐002). All participants provided written informed consent. Paraffin sections were dewaxed in xylene, rehydrated in graded ethanol, and subjected to microwave‐based antigen retrieval in citrate buffer (to boiling, then natural cooling). This process was repeated 2–3 times. After PBS washing, endogenous peroxidase activity was blocked, and goat serum was added at 37°C for 15 min to minimize nonspecific binding.

Sections were incubated overnight at 4°C with primary antibodies (COL4A1, CXCR4, MMP7; Servicebio, Wuhan), then with biotinylated secondary antibody for 2 h, followed by streptavidin‐peroxidase complex for 15 min at room temperature. A fresh DAB substrate was used to develop positive signals, and the reaction was terminated once brownish‐yellow staining appeared on a light background. Slides were counterstained with hematoxylin, dehydrated, cleared in xylene, and mounted with neutral balsam. Images were acquired by light microscopy, and positive staining was quantified as integrated optical density (IOD) with Image‐Pro Plus.

### 2.13. Biostatistical Analysis

All statistics used R v4.2.2; two‐group comparisons used the Wilcoxon rank‐sum test, with *p*  < 0.05 considered significant.

## 3. Results

### 3.1. Acquisition of 37 Candidate Genes

**Figure 1:**
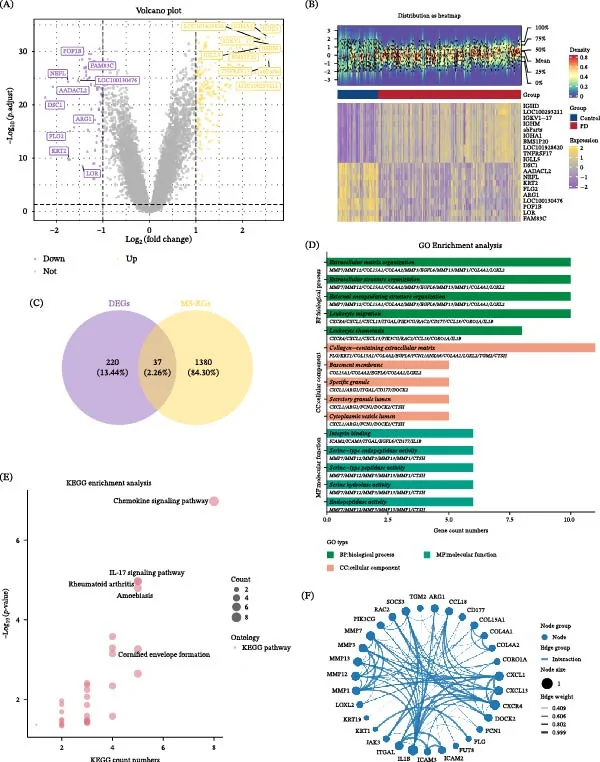
Acquisition of 37 candidate genes. (A, B) Volcano plot and heatmap presenting bulk DEGs (adj.*p*  < 0.05 and |log_2_FC| > 1). (C) Venn intersection of bulk DEGs and MS‐RGs, identifying 37 candidate genes. (D, E) GO and KEGG enrichment profiles for the 37 candidates (*p*  < 0.05). (F) PPI network of proteins encoded by the candidate genes (interaction score > 0.4).

Differential expression in GSE16134 identified 257 bulk DEGs—199 up and 58 downregulated in PD (Figure 1A,B). Overlap with MS‐RGs yielded 37 candidate genes (Figure 1C). Enrichment analysis associated the candidates with 666 GO terms, such as “extracellular matrix organization,” and 31 KEGG pathways, including “Chemokine signaling pathway” and “IL‐17 signaling pathway” (Figure 1D,E; Tables S2,S3).

The PPI network included 31 connected proteins, with CXCR4 and CXCL13 among the representative nodes (Figure 1F).

### 3.2. Identification of COL15A1, COL4A1, CXCR4, and MMP7 as Biomarkers

At the optimal lambda value of 0.0077, LASSO regression retained 17 genes (Figure 2A). RF analysis identified 10 genes, and Boruta classified 32 genes as candidate features (Figure 2B,C). Intersecting the three feature selection outputs yielded four candidate biomarkers: COL15A1, COL4A1, CXCR4, and MMP7 (Figure 2D). In GSE16134 and GSE223924, each biomarker showed higher expression in PD samples than in controls (*p*  < 0.001) (Figure 2E). ROC analysis produced AUC values of 0.895 for COL15A1, 0.881 for COL4A1, 0.887 for CXCR4, and 0.866 for MMP7, indicating favorable predictive performance (Figure 2F).

Figure 2Identification of COL15A1, COL4A1, CXCR4, and MMP7 as biomarkers. (A) LASSO cross‐validation and coefficient path plots (optimal *λ* = 0.0077). (B) RF feature ranking (ntree = 13). (C) Boruta feature‐selection output. (D) Venn diagram of four biomarkers shared by the three algorithms. (E) Biomarker expression patterns in GSE16134 and GSE223924 (*p*  < 0.05). (F) ROC evaluation of biomarker performance in GSE16134 (AUC > 0.7). The  ^∗∗∗^ indicates a statistically significant difference with a *p*‐value < 0.001 between the Control and PD groups.

**Figure:**
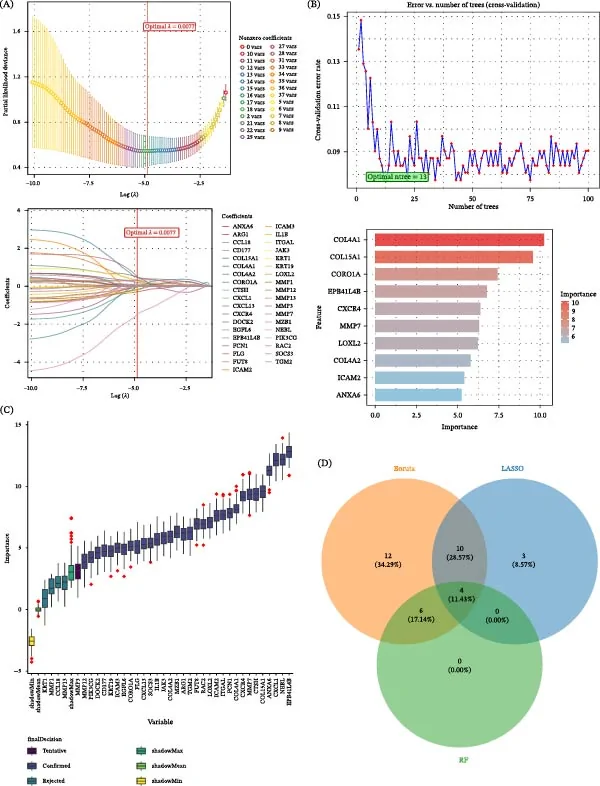

**Figure:**
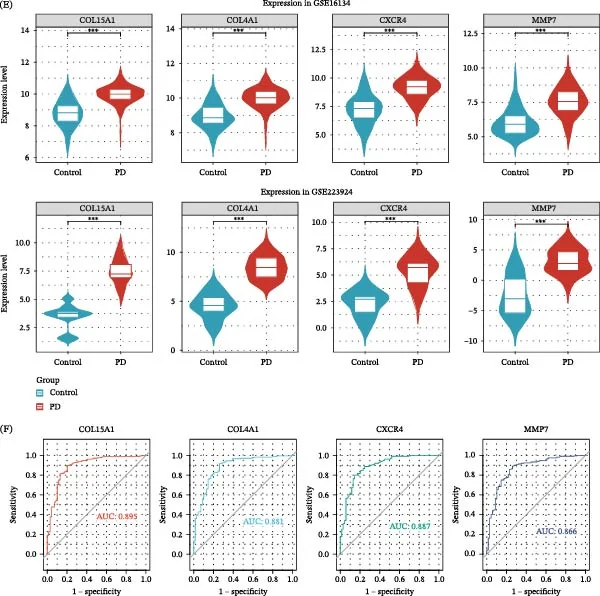

### 3.3. A Nomogram Model With Good Predictive Performance

**Figure 3:**
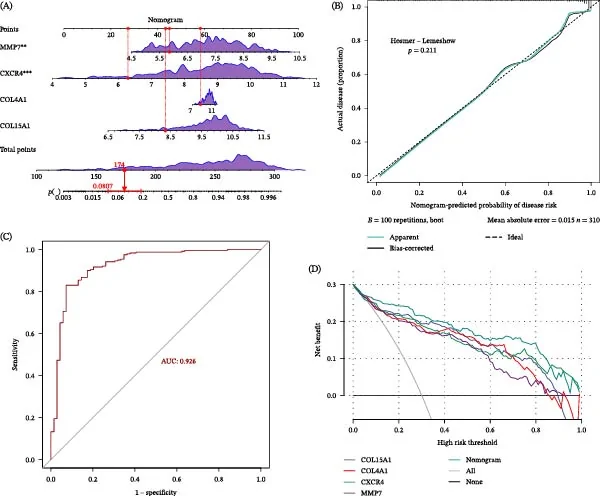
Nomogram construction and assessment. (A) Nomogram generated from the selected biomarkers. (B) Calibration curve for the nomogram (HL test *p*  = 0.211). (C) ROC curve showing overall model performance (AUC = 0.926). (D) DCA curve indicating clinical net benefit. The asterisks indicate the statistical significance of the variables in the model:  ^∗∗^ represents *p* < 0.01, and  ^∗∗∗^ represents *p* < 0.001.

The nomogram assigned a point value to the expression level of each biomarker, and the four point values were summed as a total score. This score was used to estimate the PD probability, with higher scores indicating greater predicted risk. A total score of 174, for instance, corresponded to a predicted probability of 0.0807 (Figure 3A). The calibration curve indicated acceptable agreement (*p*  = 0.211), and the integrated model achieved an AUC of 0.926 (Figure 3B,C). DCA suggested that the nomogram offered a greater clinical net benefit than single‐gene models (Figure 3D).

### 3.4. Multiple Signaling Pathways in PD

**Figure 4:**
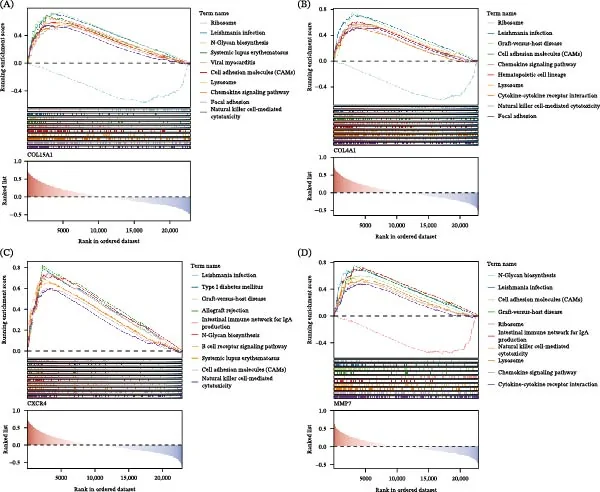
Exploration of signaling pathways. (A) Top 10 pathways enriched for COL15A1. (B) Top 10 pathways enriched for COL4A1. (C) Top 10 pathways enriched for CXCR4. (D) Top 10 pathways enriched for MMP7.

GSEA identified 80 enriched pathways for COL15A1, including “Cell adhesion molecules (CAMs)”; 73 pathways for COL4A1, including “Cytokine–cytokine receptor interaction;” 69 pathways for CXCR4, including “B cell receptor signaling pathway;" and 60 pathways for MMP7, including “Chemokine signaling pathway” (Figure 4A–D; Tables S4–S7). Common pathways included “CAMs,” “Chemokine signaling pathway,” “Natural killer cell‐mediated cytotoxicity,” and “Cytokine–cytokine receptor interaction.” These findings suggested links with immune regulation, tissue homeostasis, and inflammatory signaling in PD.

### 3.5. Association Between Biomarkers and Immune Infiltration

Stacked bar plots showed relative proportions of 22 immune subsets in PD and controls; plasma cells were more abundant in PD (Figure 5A). Fifteen subsets—neutrophils and resting natural killer (NK) cells among them—differed significantly between groups (*p*  < 0.05, Figure 5B). Spearman analysis identified positive correlations between plasma cells and all four biomarkers (COL15A1: cor = 0.58, *p*  < 0.001; COL4A1: cor = 0.48, *p*  < 0.001; CXCR4: cor = 0.52, *p*  < 0.001; MMP7: cor = 0.58, *p*  < 0.001) (Figure 5C). These results indicated that the biomarker profile was associated with immune‐cell remodeling in PD.

Figure 5Association between biomarkers and the immune microenvironment. (A) Stacked bar plot of 22 immune cell subsets. (B) Box plot presenting differentially abundant immune cell subsets (*p*  < 0.05). (C) The top five biomarker–immune‐cell correlations (|cor| > 0.30, *p*  < 0.05) are shown. The asterisks indicate the level of statistical significance for the correlation analysis:  ^∗^ *p* < 0.05,  ^∗∗^ *p* < 0.01,  ^∗∗∗^ *p* < 0.001, and  ^∗∗∗∗^ *p* < 0.0001.

**Figure:**
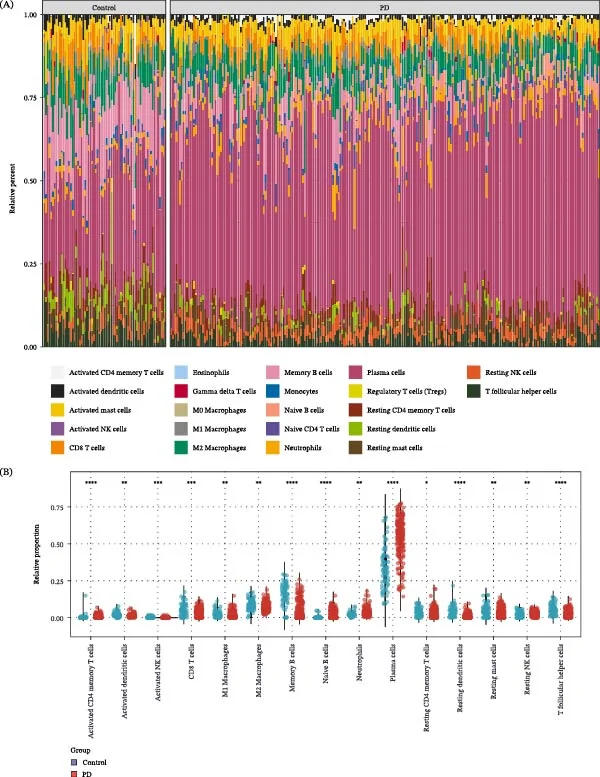

**Figure:**
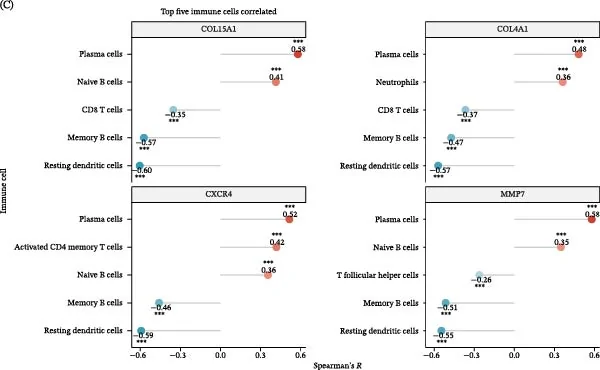

### 3.6. Multiperspective Investigation of Biomarkers’ Functions

Subcellular localization prediction suggested that COL15A1 was mainly distributed in the cytoplasm, endomembrane system, endoplasmic reticulum, and other intracellular anatomical structures. This pattern implied possible involvement in protein synthesis, folding, transport, and matrix assembly. CXCR4 was primarily predicted at the cell surface, plasma membrane, cell margin, and leading edge, consistent with a membrane receptor or signaling function. Predicted localization data were not obtained for the other two biomarkers (Figure 6A). Chromosomal mapping placed COL15A1, COL4A1, CXCR4, and MMP7 on chromosomes 9, 13, 2, and 11, respectively (Figure 6B).

Figure 6Multiperspective investigation of biomarker functions. (A) Predicted subcellular localization of COL15A1 and CXCR4. (B) Chromosomal positions of COL15A1, COL4A1, CXCR4, and MMP7. (C) lncRNA‐key miRNA–mRNA regulatory network. (D) TF‐mRNA regulatory network. (E) Drug/compound–mRNA network.

**Figure:**
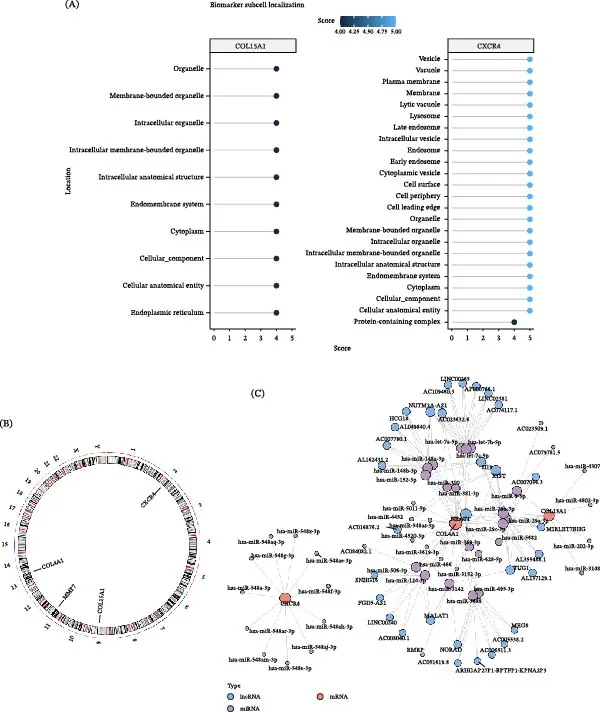

**Figure:**
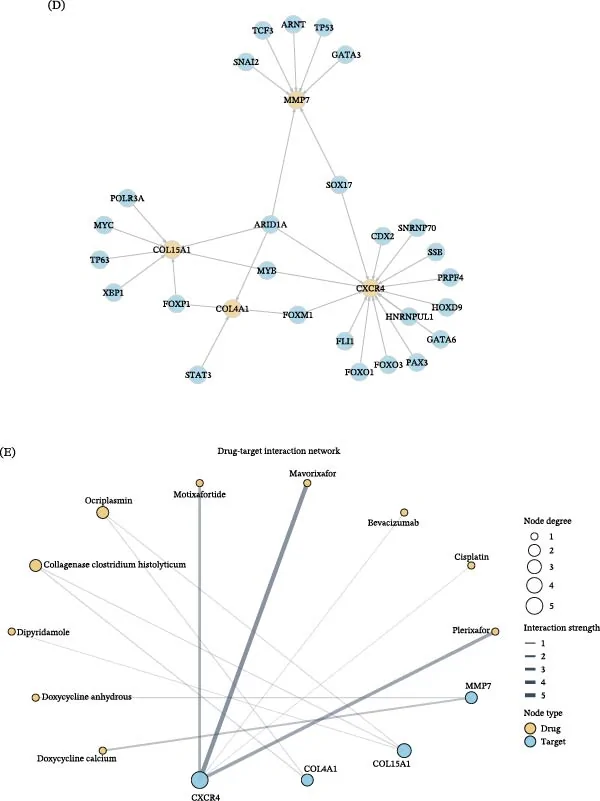

Prediction analyses yielded 42 key miRNAs, including hsa‐miR‐124–3 p, and 35 lncRNAs, including TUG1. The resulting regulatory axes included MALAT1‐hsa‐miR‐124–3 p‐COL4A1 and MALAT1‐hsa‐miR‐3142‐COL4A1 (Figure 6C). Five TFs, including FOXP1, FOXM1, MYB, ARID1A, and SOX17, were linked with multiple biomarkers. Representative TF‐mRNA pairs included SOX17‐MMP7 and SOX17‐CXCR4 (Figure 6D).

### 3.7. Drugs/Compounds Associated With Biomarkers

Drug/compound prediction revealed three agents associated with COL15A1, including dipyridamole; two agents associated with COL4A1, including ocriplasmin; five agents associated with CXCR4, including plerixafor; and two agents associated with MMP7, including doxycycline calcium (Figure 6E). Among them, dipyridamole has been reported to attenuate PD‐related tissue injury by regulating M1 macrophage polarization.

### 3.8. Annotation of 13 Cell Types

After stringent QC of GSE171213, 33,079 cells and 36,412 genes remained for single‐cell analysis (Figure S1A). A total of 2000 HVGs were selected, and the top 10 HVGs, including HBB, were labeled in the visualization (Figure S1B). Dimensionality reduction by PCA retained the top 20 PCs, capturing major variation for downstream analyses (Figure S1C).

**Figure 7:**
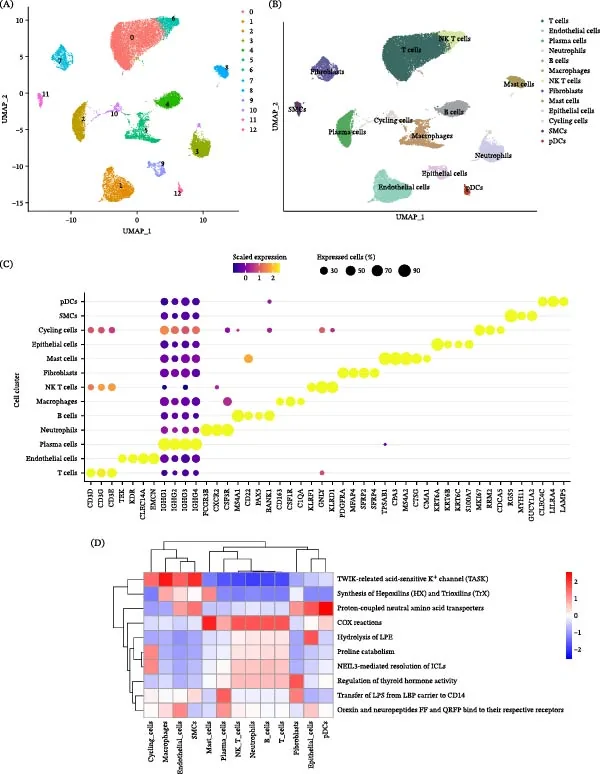
Single‐cell analysis. (A) UMAP/clustering results showing 13 cell clusters. (B) Annotation of the 13 cell types. (C) Marker‐gene expression patterns across cell types. (D) Pathways enriched across different cell types.

Unsupervised clustering divided the cells into 13 clusters (Figure 7A). According to canonical marker genes, these clusters were annotated as 13 cell types: T cells, endothelial cells, plasma cells, neutrophils, B cells, macrophages, NKT cells, fibroblasts, mast cells, epithelial cells, cycling cells, SMCs, and pDCs (Figure 7B). Marker expression across the annotated cell types was displayed using a bubble plot (Figure 7C).

Cell‐level enrichment analysis indicated that several cell types were enriched in the “TWIK‐related acid‐sensitive K^+^ channel (TASK)” pathway (Figure 7D). This pattern suggested that K^+^ channels may be involved in regulating cellular functions and physiological processes in PD.

### 3.9. Pseudotemporal Trajectory Analysis of Key Endothelial Cells

Cell composition analysis: control samples had higher T‐ and B‐cell fractions, while PD samples showed larger T‐cell and endothelial proportions (Figure 8A). Four cell types—T cells, endothelial cells, epithelial cells, and SMCs—differed significantly between groups (Figure 8B). Endothelial cells exhibited high COL4A1 and COL15A1 expression and were therefore selected as key cells for subsequent analyses (*p*  < 0.05) (Figure 8C).

Figure 8Pseudotemporal trajectory analysis of key endothelial cells. (A) Cell type proportions in PD and control samples. (B) Cell types showing significant proportional differences between groups (*p*  < 0.05). (C) Biomarker expression across cell types. (D) Four endothelial cell subclusters. (E) Pseudotime, state, cluster, and group distributions along endothelial cell trajectories. (F) Biomarker expression dynamics during endothelial cell differentiation. The asterisk indicate the level of statistical significance for the correlation analysis:  ^∗^ *p* < 0.05.

**Figure:**
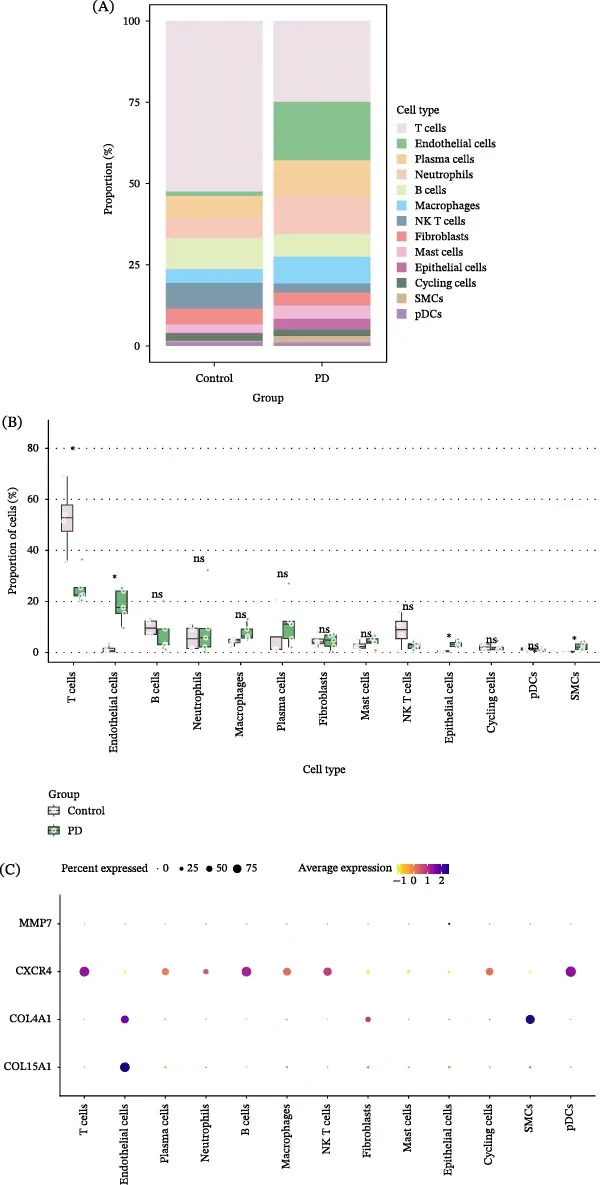

**Figure:**
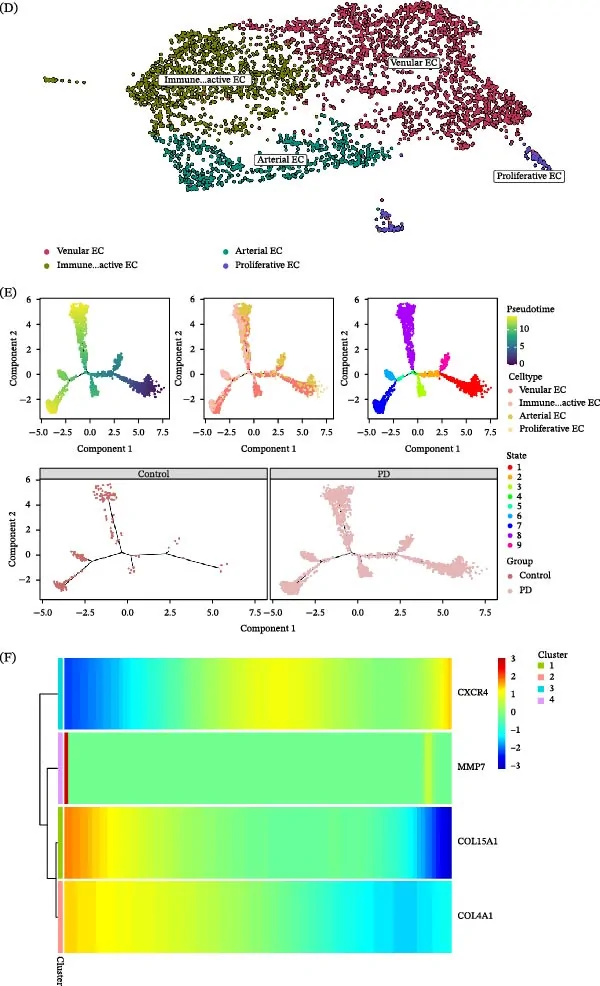

Endothelial cells were further stratified into four subclusters: venular endothelial cells, immune‐active endothelial cells, arterial endothelial cells, and proliferative endothelial cells (Figure 8D). Pseudo‐time analysis showed that endothelial cell trajectories progressed from right to left. Proliferative endothelial cells were mainly located at the early pseudotime. The trajectory was separated into nine stages, with stage 1 representing the early phase. PD‐derived cells showed a broader distribution along the trajectory (Figure 8E). Biomarker dynamics indicated that CXCR4 was expressed mainly during the middle‐to‐late stages, MMP7 appeared transiently in the early stage, and COL15A1 and COL4A1 were highly active early in the trajectory (Figure 8F). Overall, endothelial cells from PD samples displayed slightly greater differentiation‐related activity, implying a possible association with PD progression.

### 3.10. Exploring Cell Functions From Multiple Perspectives

CellChat analysis indicated stronger endothelial cell communication with fibroblasts and SMCs in PD than in controls, whereas endothelial‐epithelial communication was absent in PD. The communication intensity between endothelial cells and neutrophils was also increased in PD samples. Given the close association between neutrophils and inflammatory responses, endothelial cells may participate in periodontal inflammation and PD progression (Figure 9A). In both PD and control groups, the highest communication probability between endothelial cells and neutrophils occurred through the ANXA1‐FPR1 ligand–receptor pair. ANXA1, secreted by epithelial/endothelial/macrophage and other cells, regulates inflammation; FPR1, primarily expressed on neutrophils, transduces ANXA1 signaling (Figure 9B).

Figure 9Multiperspective exploration of cell functions. (A) Intercellular communication networks based on count and weight. (B) Ligand–receptor profiles involving endothelial cells. (C) Cell‐cycle distribution in endothelial cells. (D) Cell‐cycle phase proportions across cell types in PD and control groups. (E) Differences in S‐phase and G2/M‐phase scores across cell types between groups (*p*  < 0.05). (F) Metabolic pathways enriched in endothelial cells. The asterisks indicate the level of statistical significance for the correlation analysis:  ^∗^ *p* < 0.05,  ^∗∗^ *p* < 0.01, and  ^∗∗∗^ *p* < 0.001.

**Figure:**
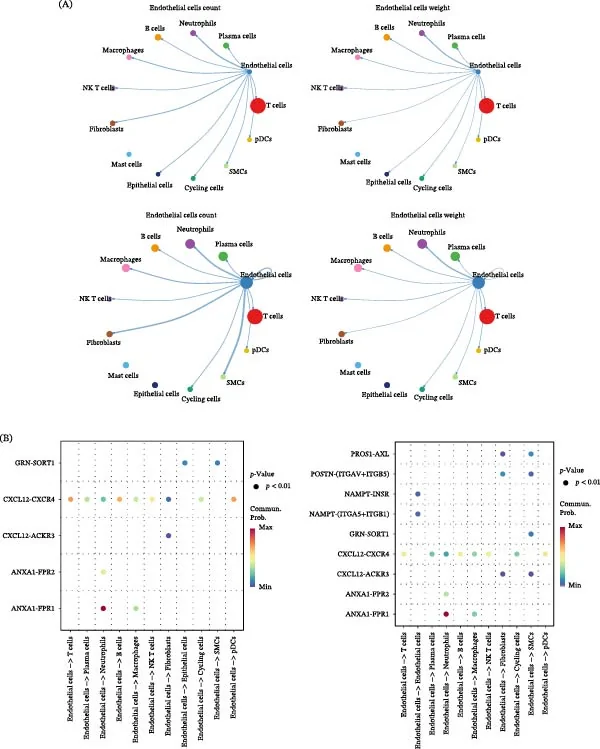

**Figure:**
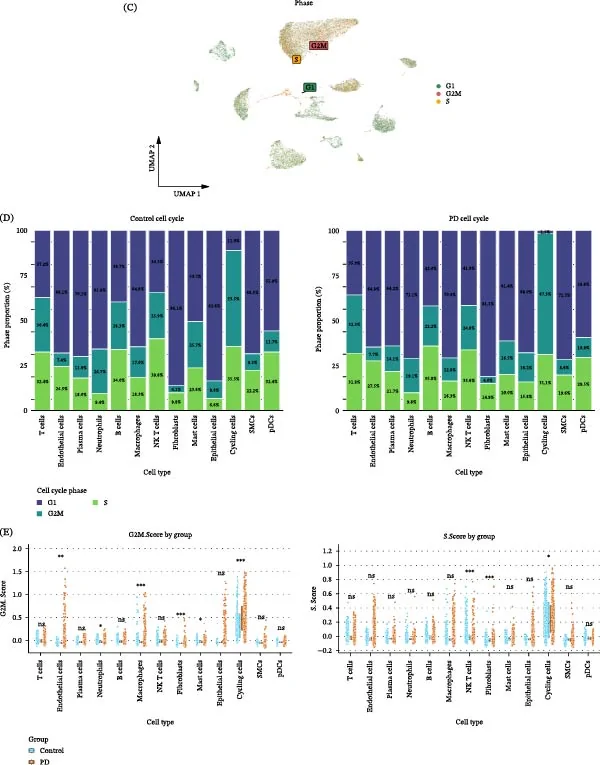

**Figure:**
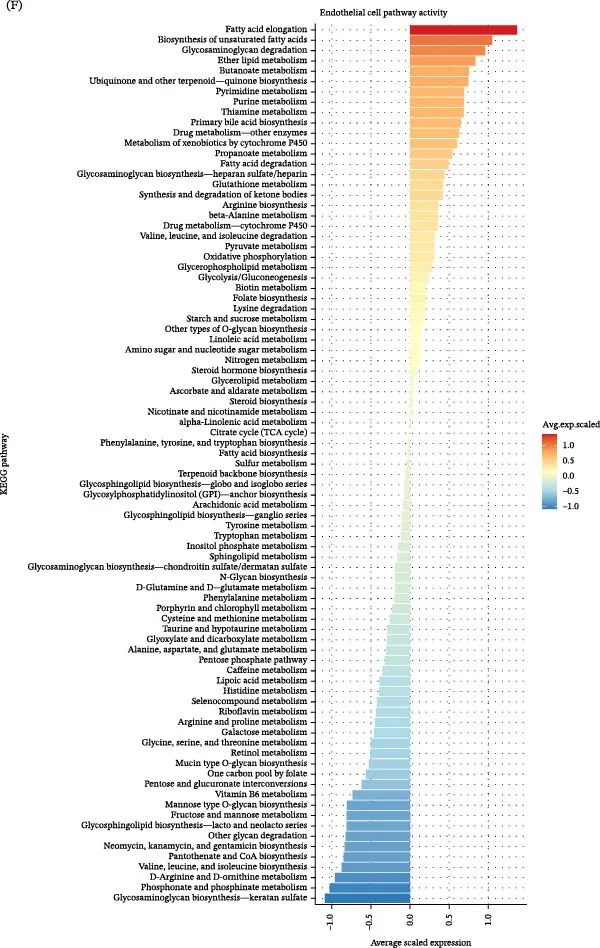

Most endothelial cells were assigned to the G1 phase (Figure 9C). A relatively high G1‐phase proportion was also observed in most cell types except cycling cells (Figure 9D). G2M scores differed significantly between PD and control groups in endothelial cells, macrophages, cycling cells, and fibroblasts. S‐phase scores differed in NKT cells, cycling cells, and fibroblasts (Figure 9E). These differences further indicated an altered proliferative activity across cell populations.

Metabolic analysis revealed elevated endothelial‐cell activity in “Fatty acid elongation” and “Biosynthesis of unsaturated fatty acids” (Figure 9F), suggesting that fatty acid metabolism may drive PD progression via inflammatory factor modulation.

### 3.11. Upregulation of COL4A1, CXCR4, and MMP7 Expression in PD Tissues

**Figure 10:**
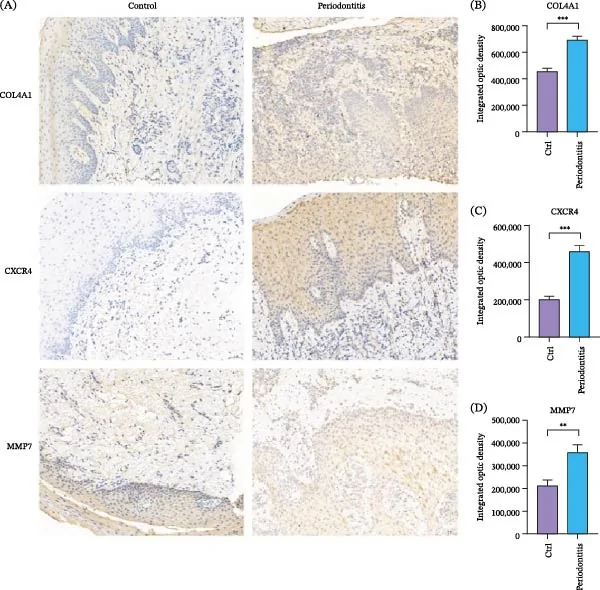
Upregulation of COL4A1, CXCR4, and MMP7 expression in periodontitis tissues. (A) Representative IHC staining images showing COL4A1, CXCR4, and MMP7 protein expression in control and periodontitis tissues. (B–D) IOD quantification of COL4A1 (B), CXCR4 (C), and MMP7 (D) based on IHC staining. The asterisks indicate the level of statistical significance for the correlation analysis:  ^∗^ *p* < 0.05,  ^∗∗^ *p* < 0.01,  ^∗∗∗^ *p* < 0.001, and  ^∗∗∗∗^ *p* < 0.0001.

To evaluate protein‐level changes related to PD, IHC was performed to assess COL4A1, CXCR4, and MMP7 in control and PD tissues. As shown in Figure 10A, control tissues displayed weak or minimal staining for all three proteins. In contrast, PD tissues showed stronger brownish‐yellow staining, indicating increased protein expression.

IOD quantification matched staining observations: COL4A1 was elevated in PD tissues versus controls (*p*  < 0.001, Figure 10B), and CXCR4 was also significantly increased in PD (*p*  < 0.001, Figure 10C). MMP7 expression showed a significant elevation as well (*p*  < 0.01; Figure 10D).

## 4. Discussion

MS is a central physical feature of the ECM and may contribute to PD by influencing periodontal tissue cells and inflammatory activity. MS has been shown to regulate proinflammatory responses and ECM synthesis in human gingival fibroblasts (hGFs) through the YAP/NF‐κB pathway. Soft matrices may aggravate gingival injury, whereas stiff matrices may support tissue homeostasis [41]. Beyond serving as a mechanical cue, MS may also be converted into intracellular stress signals. Recent work has emphasized the role of the integrated stress response (ISR) in periodontal inflammation. Therefore, changes in MS may activate ISR‐related processes and connect the physical microenvironment with the inflammatory phenotype of PD. In the present study, four biomarkers, COL15A1, COL4A1, CXCR4, and MMP7, were identified and validated from GEO datasets using multiple bioinformatic strategies, including differential expression analysis. We further investigated their potential mechanisms through GSEA and immune cell infiltration profiling, providing new perspectives for PD diagnosis and therapeutic exploration.

CXCR4, a seven‐transmembrane G protein‐coupled chemokine receptor, participates in diverse inflammation‐related processes [42]. Its relationship with MS has been experimentally supported as a stiff matrix (28 kPa) can markedly increase CXCR4 protein expression [43]. CXCR4 is also connected with the pathological progression of PD. In PD, CXCR4 signaling has been reported to mediate *Porphyromonas gingivalis*‐induced alveolar bone resorption, and pathway blockade can attenuate bone loss [44]. CXCR4 may also participate in PD pathology through the activation of the PI3K/Akt/NF‐κB signaling pathway [45].

The CXCR4/CXCL12 axis may further influence PD progression by regulating neutrophil dynamics. Neutrophil accumulation in periodontal tissues is a characteristic feature of PD and reflects a strong host immune response against periodontal pathogens [46]. Activated neutrophils release ROS, proteases, and antimicrobial peptides. ROS drive oxidative tissue damage, while proteases—including MMPs, elastase, and cathepsins—degrade ECM components and exacerbate tissue destruction. Neutrophils also secrete proinflammatory cytokines that amplify local immune responses and aggravate periodontal damage [47, 48]. In our study, CXCR4 and other biomarkers were upregulated in PD and were involved in the “Chemokine signaling pathway.” The PD group also showed a higher neutrophil proportion. We therefore hypothesize that increased CXCR4 expression may activate chemokine signaling, regulate neutrophil dynamics, increase neutrophil infiltration, alter MS, and promote periodontal tissue destruction. Further experimental validation is necessary to clarify this mechanism.

Previous studies have shown that matrix rigidity can increase SOX2 expression and maintain stemness in hepatocellular carcinoma cells [49]. Conversely, a soft matrix can also induce SOX2 expression and enhance the antiapoptotic capacity in laryngeal squamous cell carcinoma cells [50]. In the present TF analysis, SOX17 was predicted as an upstream regulator of CXCR4. SOX17 belongs to the same gene family as SOX2 [51, 52]. On this basis, MS may regulate CXCR4 by altering SOX17 expression or activity. Such regulation could activate downstream pathways, including the “Chemokine signaling pathway,” strengthen inflammatory responses, and contribute to PD progression. This interpretation remains inferential. Because the supporting evidence is drawn from tumor models and from SOX2 rather than SOX17, direct validation in PD‐specific systems is required.

COL15A1 encodes type XV collagen and functions as an important structural component of the ECM. By forming a cross‐linked network with other collagens and proteoglycans, it helps maintain ECM compactness and spatial stability. Loss of Col15a1 disrupts ECM integrity and weakens the physical barrier function of the matrix [53]. In periodontal tissues, COL15A1 has been implicated in maintaining the mechanical support of the ECM. Abnormal expression may compromise the periodontal ligament‐alveolar bone interface, accelerate attachment loss, and promote PD progression [54]. NK cells can directly recognize *Fusobacterium nucleatum* through the surface natural cytotoxicity receptor NKp46, thereby activating NK cell‐mediated cytotoxicity. This activation induces substantial secretion of proinflammatory cytokines, including IFN‐γ and TNF‐α, and ultimately worsens inflammation and tissue destruction in PD [55]. In addition, NK cells were enriched in PD lesions. These cells release proinflammatory cytokines, recruit neutrophils and macrophages, amplify inflammatory cascades, and aggravate clinical phenotypes such as gingival redness and bleeding [56, 57].

In this study, COL15A1 was markedly increased in PD and was linked to the “Natural killer cell‐mediated cytotoxicity” pathway. Resting NK cells also showed significant infiltration in the PD group. These findings support the hypothesis that elevated COL15A1 may be associated with activation of NK cell‐mediated cytotoxicity, thereby increasing proinflammatory cytokine release and facilitating PD progression. This proposed relationship requires direct experimental testing.

Overall, the four biomarkers identified here appear closely related to MS regulation. COL15A1 and COL4A1 are structural collagens that directly influence ECM integrity and mechanical properties [58, 59]. Their upregulation in PD may reflect compensatory or pathological remodeling of MS. CXCR4 expression has been shown to increase on rigid matrices (28 kPa). MMP7, as a matrix metalloproteinase, may serve as both a regulator and an indicator of ECM stiffness because it degrades matrix components and can be induced by mechanical stress [60, 61]. Together, these biomarkers may form a stiffness‐sensitive network that promotes PD through inflammatory chemotaxis and immune cell infiltration.

Based on the Drug‐Gene Interaction Database, dipyridamole was predicted to interact with COL15A1. In a rat model of PD, dipyridamole reduced alveolar bone loss, decreased proinflammatory cytokine protein expression, suppressed osteoclastogenesis, and inhibited macrophage infiltration and M1 macrophage polarization in gingival tissues [62]. In addition, lipopolysaccharide (LPS)‐stimulated macrophages show increased proinflammatory cytokine secretion as MS rises [63]. Our results showed that the M1 macrophage expression was increased in PD. Considering the predicted relationship between COL15A1 and dipyridamole, we hypothesize that dipyridamole may interact with COL15A1‐related mechanisms to maintain ECM structural stability, alleviate abnormal MS, reduce M1 macrophage polarization, and mitigate inflammation.

Single‐cell analysis in this study identified endothelial cells as key regulatory cells. Increased endothelial permeability can facilitate leukocyte transendothelial migration and promote tissue inflammation [64], and PD‐related pathogenic factors also increase vascular permeability [65]. *P. gingivalis* infection markedly suppresses plasminogen activator inhibitor‐1 (PAI‐1) expression in human endothelial cells. Degradation of PAI‐1 impairs endothelial permeability and function through low‐density lipoprotein receptor‐related proteins [66]. Endothelial cells from PD patients also show increased phosphatidylserine (PS) exposure. Suppression of inflammatory factors can significantly reduce the PS expression in endothelial cells [67].

Endothelial fatty acid uptake/transport sustains membrane synthesis, signaling, ATP production, protein modification, and metabolic gene regulation [68]. Resting cells express fatty acid oxidation genes and preserve redox balance via NADPH regeneration; mouse CPT1 deficiency (rate‐limiting for FAO) drives endothelial dysfunction through oxidative stress [69]. In PD, fatty acid synthase (FASN)‐mediated lipogenesis regulates endothelial permeability and contributes to bone loss [70].

In the present analysis, endothelial cells, as key cells, showed high activity in fatty acid‐related pathways, including “Fatty acid elongation” and “Biosynthesis of unsaturated fatty acids.” We hypothesize that the increased proportion of endothelial cells in PD may regulate these pathways, enhance endothelial permeability, and promote bone loss. Increased endothelial permeability may further intensify tissue inflammation, stimulate inflammatory factor secretion, and recruit neutrophils, macrophages, and other immune cells, thereby amplifying the local inflammatory cascade.

This study identified four core biomarkers potentially linked to MS regulation: COL15A1, COL4A1, CXCR4, and MMP7. These biomarkers were highly expressed in PD tissues and showed good diagnostic predictive value, with all AUC values > 0.7. They were also involved in inflammatory regulatory pathways, including the chemokine signaling pathway and NK cell‐mediated cytotoxicity. Fifteen immune cell types, including neutrophils and resting NK cells, differed between groups and were strongly associated with the biomarkers. Network interactions (MALAT1–miR‐124‐3p–COL4A1 and SOX17–CXCR4) and dipyridamole were identified. Endothelial cells were key single‐cell types with elevated fatty‐acid elongation/unsaturated biosynthesis, providing a basis for MS‐gene research in PD.

Several limitations should be noted. First, this study was based mainly on public database resources, which lack detailed clinical information. Bioinformatic analyses can identify correlations but cannot establish biological causality. Second, although IHC was added to validate increased protein expression of candidate genes, this represents expression‐level evidence only. Functional assays and mechanical measurements have not yet been performed. Third, MS was not directly measured in periodontal tissues, and mechanistic inference relied largely on previous studies and computational predictions. Single‐cell RNA‐seq data also lacked mechanical measurement information, and the analysis of cell–matrix interactions depended primarily on transcriptomic correlations. Finally, drug prediction was based entirely on computational modeling and cannot confirm the pharmacological efficacy or direct target binding.

Future studies should evaluate candidate gene expression in independent PD cohorts. In vitro models with tunable MS should also be combined with mechanical testing to assess how different stiffness conditions affect gene expression and immune pathways. Gene overexpression or knockdown experiments are needed to determine the functional roles of candidate genes in cell migration, inflammatory cytokine secretion, and metabolic activity. In addition, the therapeutic effects of predicted drugs, including dipyridamole, and their regulatory relationships with candidate genes should be validated in animal models. Only after such comprehensive validation can the biomarker or therapeutic potential of these genes in PD be more rigorously assessed.

## Funding

No funding was received for this manuscript.

## Disclosure

All content, data analysis, experimental interpretation, and scientific conclusions were independently completed, reviewed, and verified by the authors to ensure full scientific accuracy and integrity.

## Ethics Statement

The study was conducted in accordance with the Declaration of Helsinki and approved by the Ethics Committee of The First Affiliated Hospital of Zhengzhou University (Protocol Code 2023‐KY‐1160‐002 and date of approval: November 21, 2023). Written informed consent has been obtained from the patient.

## Conflicts of Interest

The authors declare no conflicts of interest.

## Supporting Information

Additional supporting information can be found online in the Supporting Information section.

## Supporting information


**Supporting Information** Figure 1: Single‐cell analysis. (A) nFeature RNA, nCount RNA, and percent.mt before and after quality control. (B) Acquisition of highly variable genes (HVGs). (C) Selection of usable principal components (PCs). Table 1: List of 1417 MS‐RGs. Table 2: GO enrichment summary table for candidate genes. Table 3: KEGG enrichment summary table for candidate genes. Table 4: Detailed enrichment results of COL15A1. Table 5: Detailed enrichment results of COL4A1. Table 6: Detailed enrichment results of CXCR4. Table 7: Detailed enrichment results of MMP7.

## Data Availability

The data that support the findings of this study are openly available in Gene Expression Omnibus (GEO) at https://www.ncbi.nlm.nih.gov/geo/, Reference Numbers GSE16134, GSE223924, and GSE171213.
