# Supplementary material for: Exploring Matrix Stiffness‐Related Gene in Periodontitis: A Comprehensive Multidataset Analysis
Source: Mediators Inflamm. 2026 Aug 26;2026:2009746. doi: 10.1155/mi/2009746 (PMC13507932; doi:10.1155/mi/2009746)
Supplement: Supplementary file 1 [file MI-2026-2009746-s001.zip › Supplementary Fig. 1A.vlnplot_before_qc.pdf]

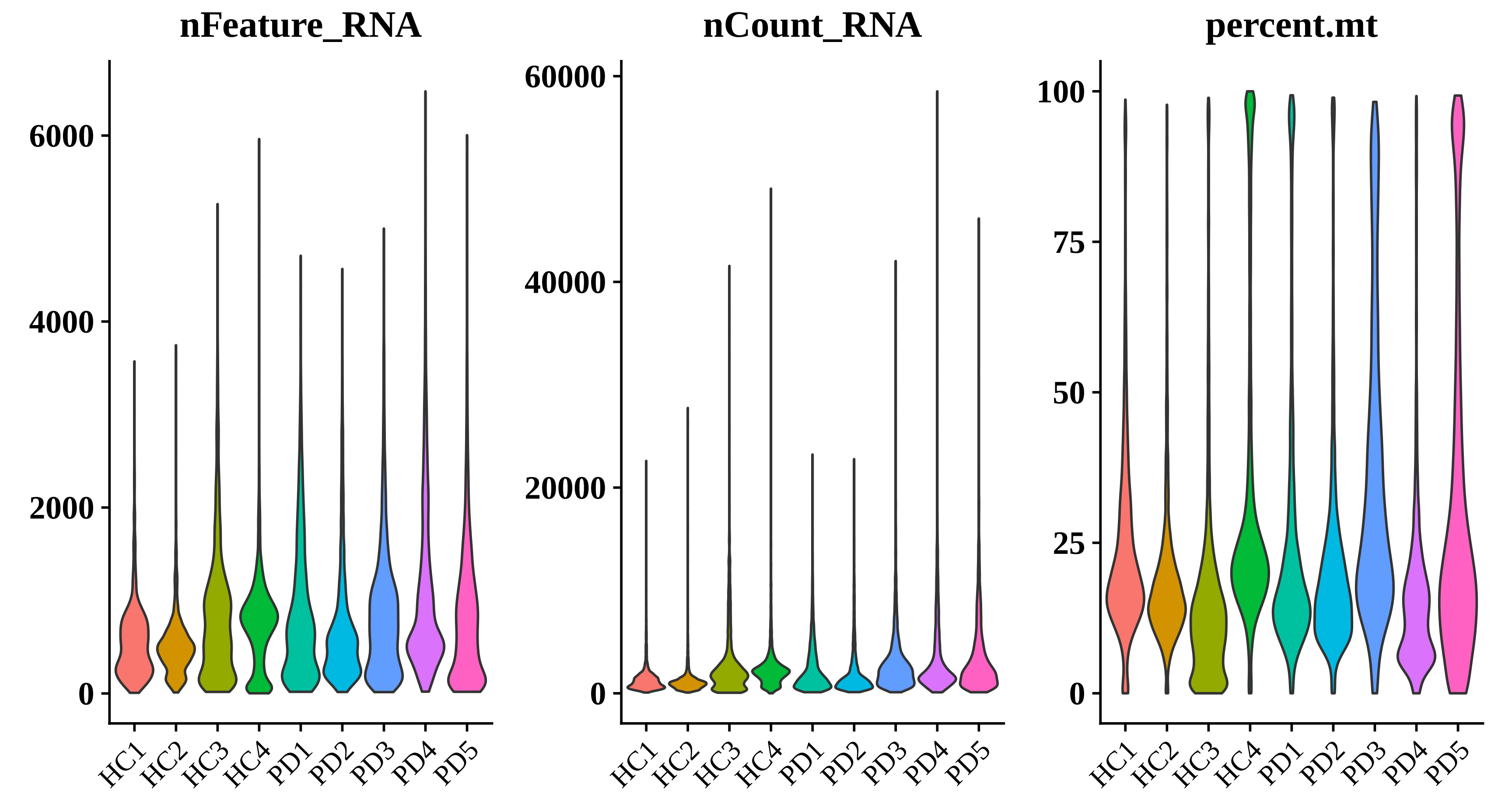

nFeature_RNA
nCount_RNA
percent.mt
0
2000
4000
6000
0
20000
40000
60000
0
25
50
75
100
HC1
HC2
HC3
HC4
PD1
PD2
PD3
PD4
PD5
