# Supplementary material for: Exploring Matrix Stiffness‐Related Gene in Periodontitis: A Comprehensive Multidataset Analysis
Source: Mediators Inflamm. 2026 Aug 26;2026:2009746. doi: 10.1155/mi/2009746 (PMC13507932; doi:10.1155/mi/2009746)
Supplement: Supplementary file 1 [file MI-2026-2009746-s001.zip › Supplementary Fig. 1B.pdf]

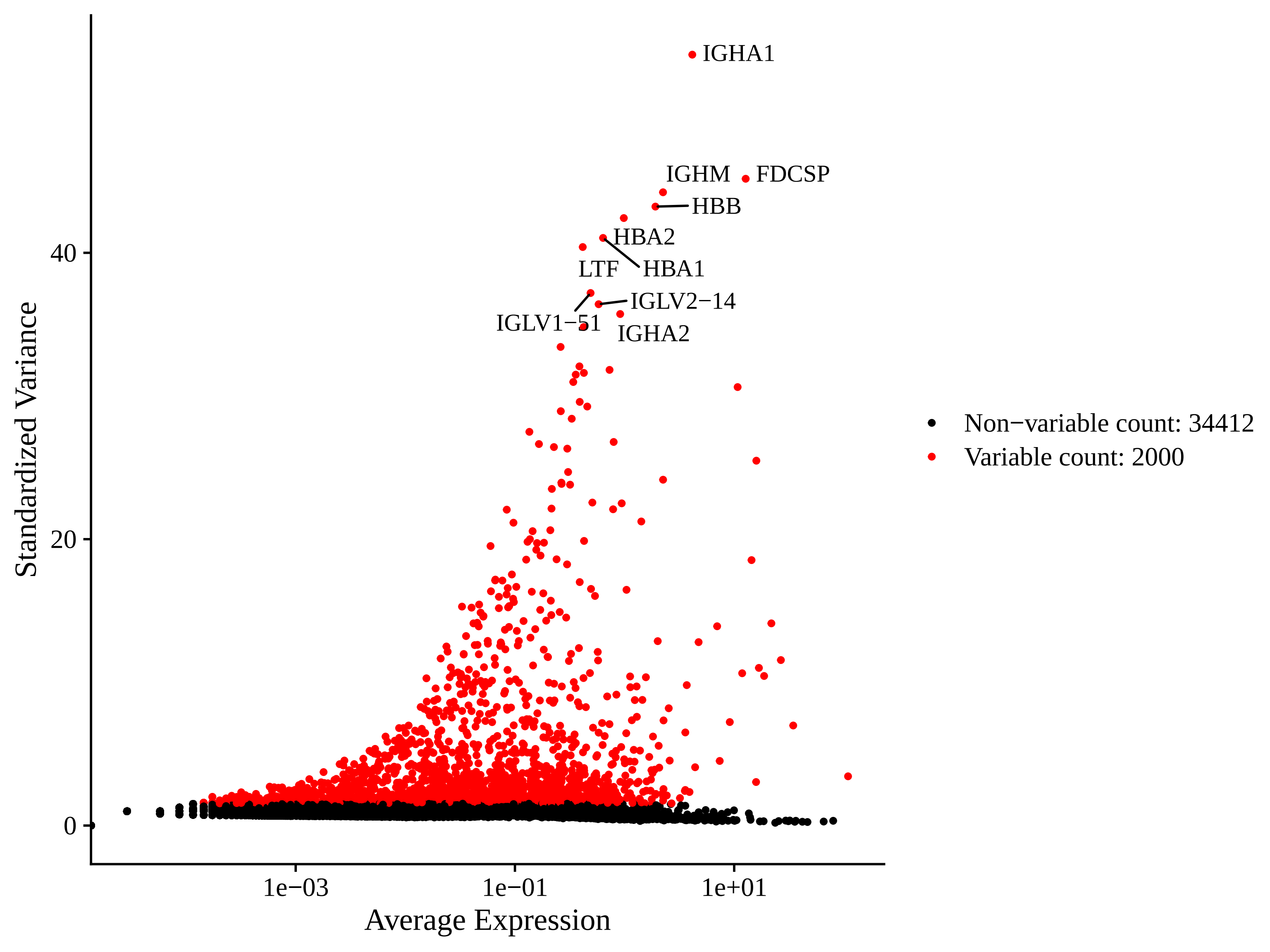

IGHA1
IGHM
FDCSP
HBB
HBA2
HBA1
LTF
IGLV2−14
IGLV1−51
IGHA2
Standardized Variance
40
20
0
1e−03
1e−01
1e+01
Average Expression
Non−variable count: 34412
Variable count: 2000
