# Supplementary material for: Exploring Matrix Stiffness‐Related Gene in Periodontitis: A Comprehensive Multidataset Analysis
Source: Mediators Inflamm. 2026 Aug 26;2026:2009746. doi: 10.1155/mi/2009746 (PMC13507932; doi:10.1155/mi/2009746)
Supplement: Supplementary file 1 [file MI-2026-2009746-s001.zip › Supplementary Fig. 1C.pca_cluster.pdf]

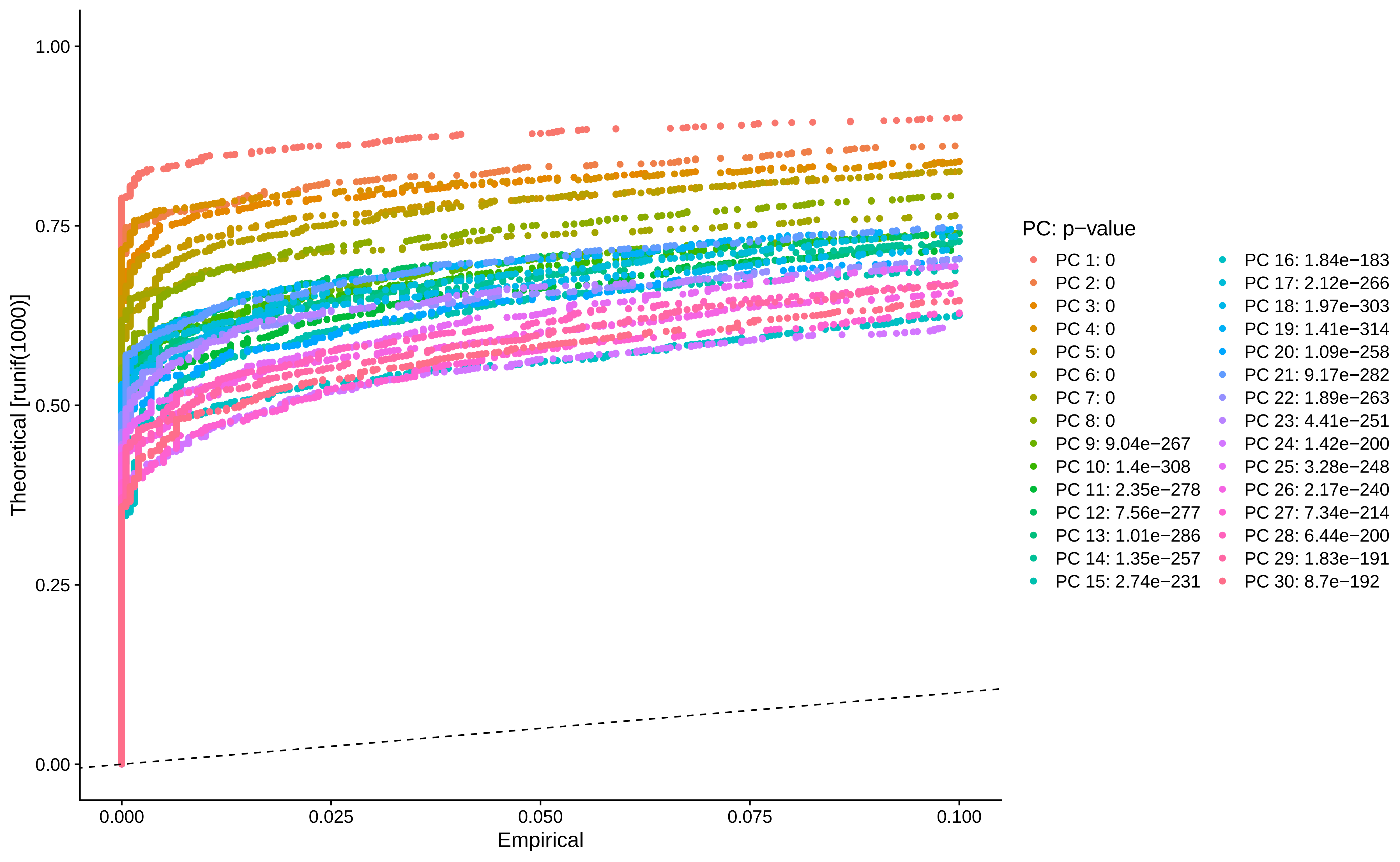

Theoretical [runif(1000)]
Empirical
0.00
0.25
0.50
0.75
1.00
0.000
0.025
0.050
0.075
0.100
PC: p−value
PC 1: 0
PC 2: 0
PC 3: 0
PC 4: 0
PC 5: 0
PC 6: 0
PC 7: 0
PC 8: 0
PC 9: 9.04e−267
PC 10: 1.4e−308
PC 11: 2.35e−278
PC 12: 7.56e−277
PC 13: 1.01e−286
PC 14: 1.35e−257
PC 15: 2.74e−231
PC 16: 1.84e−183
PC 17: 2.12e−266
PC 18: 1.97e−303
PC 19: 1.41e−314
PC 20: 1.09e−258
PC 21: 9.17e−282
PC 22: 1.89e−263
PC 23: 4.41e−251
PC 24: 1.42e−200
PC 25: 3.28e−248
PC 26: 2.17e−240
PC 27: 7.34e−214
PC 28: 6.44e−200
PC 29: 1.83e−191
PC 30: 8.7e−192
