# Supplementary material for: Exploring Matrix Stiffness‐Related Gene in Periodontitis: A Comprehensive Multidataset Analysis
Source: Mediators Inflamm. 2026 Aug 26;2026:2009746. doi: 10.1155/mi/2009746 (PMC13507932; doi:10.1155/mi/2009746)
Supplement: Supplementary file 1 [file MI-2026-2009746-s001.zip › Supplementary Fig. 1C.pca_sd.pdf]

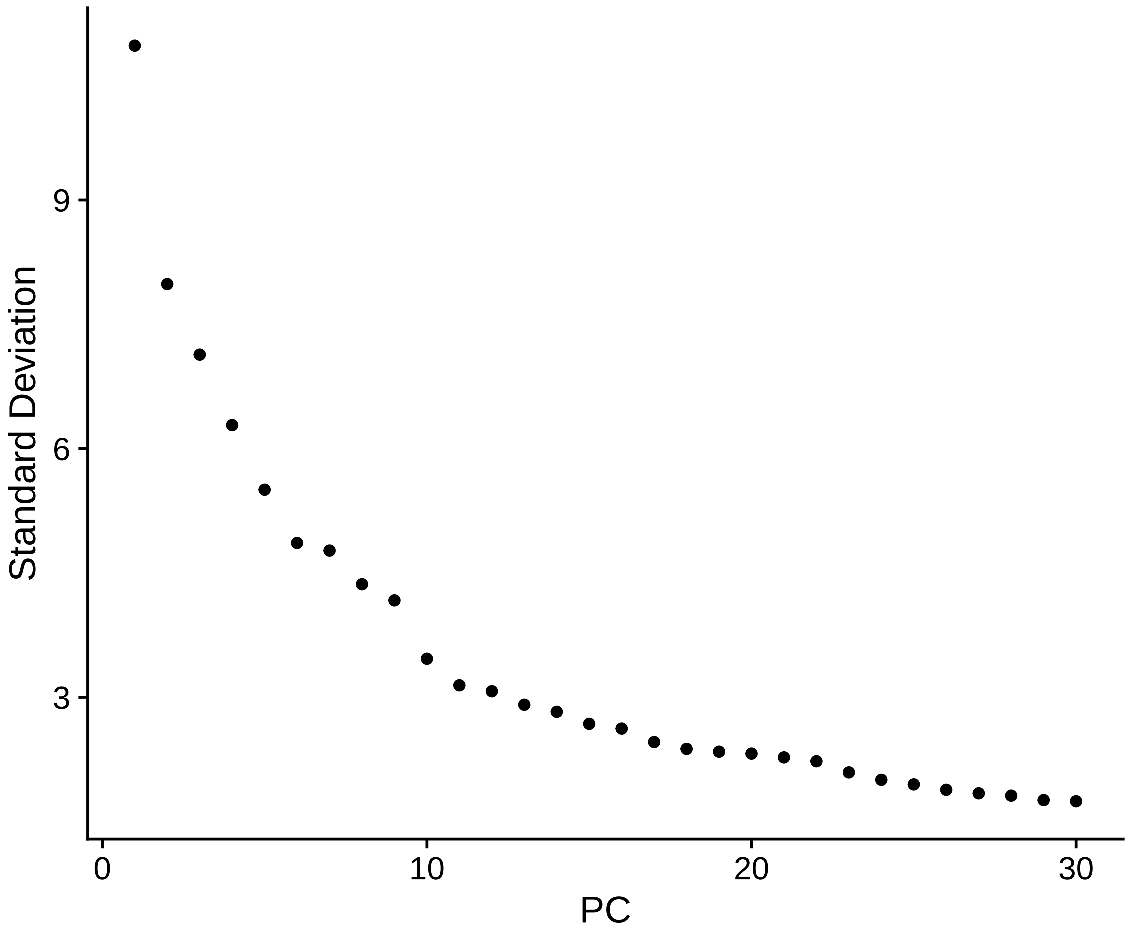

Standard Deviation
9
6
3
0
10
20
30
PC
